# Major Phytochemical as *γ*-Sitosterol Disclosing and Toxicity Testing in Lagerstroemia Species

**DOI:** 10.1155/2017/7209851

**Published:** 2017-01-16

**Authors:** Prapaparn Sirikhansaeng, Tawatchai Tanee, Runglawan Sudmoon, Arunrat Chaveerach

**Affiliations:** ^1^Department of Biology, Faculty of Science, Khon Kaen University, Khon Kaen, Thailand; ^2^Genetics and Environmental Toxicology Research Group, Khon Kaen University, Khon Kaen, Thailand; ^3^Faculty of Environment and Resource Studies, Mahasarakham University, Maha Sarakham, Thailand; ^4^Faculty of Law, Khon Kaen University, Khon Kaen, Thailand

## Abstract

Medicinal plants in genus* Lagerstroemia *were investigated for phytochemical contents by GC-MS and HPLC with ethanol and hexane extracts and their toxicity MTT and comet assay on human peripheral blood mononuclear cells (PBMCs). *γ*-Sitosterol is the major component found in all species at 14.70–34.44%. All of the extracts, except for* L. speciosa* ethanol extract, showed high percentages of cell viability. The IC_50_ value, 0.24 mg/mL, of ethanol* L. speciosa* extract predicted an LD_50_ of 811.78 mg/kg, which belongs to WHO Class III of toxic chemicals. However, in-depth toxicity evaluation by the comet assay showed that the four tested species induced significant (*p* < 0.05) DNA damage in PBMCs. *γ*-Sitosterol was previously reported to possess antihyperglycemic activity by increasing insulin secretion in response to glucose. Nonetheless, consumers should consider its toxicity, and the amount of consumption should be of concern.

## 1. Introduction

*Lagerstroemia* species, including* L. speciosa*,* L. loudonii*,* L. indica*,* L. villosa*, and* L. floribunda,* were used worldwide as medicinal and ornamental plants.* L. indica* extract has been used for treating allergic diseases such as asthma due to its anti-inflammatory properties [1, 2], analgesic, antihyperglycemic, and antioxidant hepatoprotective effects [1], and antidiabetic activity by its containing corosolic acid [3].* L. speciosa* and* L. loudonii* have also been reported for their chemical constituents [4, 5].* L. speciosa* leaf extract containing corosolic acid as an active compound has been reported for diabetes treatment [6, 7]. The hypoglycemic effects of* L. speciosa* have been attributed to both corosolic acid and ellagitannins [8]. Current knowledge on the phytochemicals and pharmacology of* L. speciosa* has regarded it as a natural antidiabetes product, whose leaves contained triterpenes, tannins, ellagic acids, glycosides, and flavones [9].

Remarkably, out of all of the natural products for diabetes treatment, the* L. speciosa* species was registered as the one of the 170 medicinal plants in Thailand listed by Ministry of Public Health announcements. However, with diverse growth factors and environments in each area of the country, its chemicals should be clarified and toxicity tested, including both cytotoxicity and genotoxicity levels. Therefore, this research focuses on the information described above and includes the following four species:* L. speciosa*,* L. indica*,* L. loudonii*, and* L. villosa*.

## 2. Materials and Methods

### 2.1. Plant Materials

Leaves of* Lagerstroemia speciosa*,* L. indica*,* L. loudonii*, and* L. villosa *were collected and used to make the crude extracts by hexane and ethanol. Then, further studies on phytochemical analysis by gas chromatography-mass spectrometry (GC-MS) and high performance liquid chromatography (HPLC), cytotoxicity by 3-(4,5-dimethylthiazol-2yl)-2,5-diphenyltetrazolium bromide (MTT) assay and genotoxicity by the comet assay were performed.

### 2.2. Phytochemical Extracts

The samples were rinsed with water and air-dried until the water evaporated from the leaves. A 20 g sample was then ground into a powder, mixed with 120 mL hexane or ethanol (analytical grade), separately for 72 h. Samples were filtered through a filter paper at room temperature, and the filtrates in this step were subjected to GC-MS analysis. For further experiments with the remaining filtrates, the solvents were evaporated with a rotary evaporator (Rotavapor R-210, Buchi, Switzerland) at 800–1,000 mbar, 15°C, and 600 rpm for 2 h. Dark green, thick, viscous crude extracts were obtained. Dimethyl sulfoxide (DMSO) was added to the extracts until being completely dissolved and maintained as stock extracts at −20°C until the cytotoxicity and genotoxicity experiments were conducted.

### 2.3. Analysis of the Plant Extract Component by GC-MS

The analysis was performed using an Agilent Technologies GC 6890 N/5973 inert mass spectrometer fused with a capillary column (30.0 m × 250 *μ*m × 0.25 *μ*m). Helium gas was used as the carrier at a constant flow rate of 1 mL/min. The injection and mass-transferred line temperature was set at 280°C. The oven temperature was programmed for 70°C to 120°C at 3°C/min, held isothermally for 2 min, and then raised to 270°C at 5°C/min. A 1 *μ*L aliquot of the crude extract was injected in split mode. The relative percentage of the crude constituents was expressed as a percentage using peak area normalization. Component identification was determined by comparing the obtained mass spectra with the reference compounds in the Wiley 7N.1 library.

### 2.4. Analysis of the Plant Extract Component by HPLC

The amount of corosolic acid from* L. speciosa* (1 mg, Sigma Aldrich) was weighed and dissolved in 1 mL of ethanol for standard solution. Contents of corosolic acid from crude extracts were determined by HPLC, using Agilent Technologies 1260 Infinity, compared to the standard. The column Hypersil ODS C18, 4.0 × 250 mm, 5 Micron (Agilent) was used. The detection wavelength was 210 nm. The mobile phase consisted of two solvents: 0.1% phosphoric acid (A) and acetonitrile (B). The gradient elution was carried out by acetonitrile 55% to 100% (0–35 min). The flow rate was 1 mL/min, and 10 *μ*L of the sample was injected.

### 2.5. Isolation of Human Peripheral Blood Mononuclear Cells (PBMCs)

PBMCs were isolated from sodium heparin anticoagulated venous blood from a blood bank using Ficoll-Paque Plus (GE Healthcare), as recommended. Freshly isolated PBMCs with viability of at least 98% were used for the toxicity testing. The cells were suspended at a concentration of 10^6^ cells/mL in modified RPMI-1640 medium, with 2 mM L-glutamine and 25 mM HEPES, supplemented with 10% FBS, 5 *μ*g/mL phytohemagglutinin (PHA), 100 *μ*g/mL streptomycin, and 100 U/mL penicillin.

### 2.6. Cell Preparations, Extract Treatments, and the MTT Assay for Cytotoxicity Testing

Upon testing, the primary crude extract concentrations were serially 10-fold diluted with water, for five levels as working concentrations. The prepared cells were seeded in 96-well plates, 125 *μ*L per well. Another 12.5 *μ*L of the proper extract working concentrations was added to the corresponding wells in triplicate. The cells were incubated for 4 h in a humidified CO_2_ incubator at 37°C and 5% CO_2_. Corresponding DMSO concentrations were similarly prepared as vehicle controls. The untreated cells were used as a negative control, whereas the positive control cells were treated with UV light for 20 min.

At the end of the treatment, the plates were centrifuged at 1,500 rpm for 10 min and the medium was removed by pipetting. The MTT (Sigma, USA) was added to a final concentration of 0.5 mg/mL in a volume of 10 *μ*L per well. Then, the plates were wrapped with aluminum foil and incubated for 4 h at 37°C. After the formazan crystals were solubilized by adding 100 *μ*L DMSO to each well, the plates were left in the dark for 2–4 h. The absorbance was read at 570 nm with a microtiter plate spectrophotometer (Fluorescence microplate reader; SpectraMax M5 series, Molecular Devices). Wells containing medium and MTT without cells were used as blanks. Each concentration treatment was performed in triplicate. All values were expressed as the mean ± S.D. Cellular reduction of tetrazolium salt, 3-(4,5-dimethylthiazol-2-yl)-2,5-diphenyltetrazolium bromide (MTT), formed a violet crystal formazan through mitochondrial succinate dehydrogenase activity of the viable cells, and the violet crystal formazan was quantified following the methods of Freshney [10]. Percentage of cell viability was calculated using the equation (cell viability (%) = average viable of treated cells/average viable of negative control cells × 100) to reveal the cytotoxicity of the plant extracts. Doses inducing 50% inhibition of cell viability (IC_50_ value) were determined by plotting a graph of the extract concentration against the cell viability. The IC_50_ value was used for the LD_50_ calculation [11] to release hazardous levels, according to the World Health Organization [12].

### 2.7. Genotoxicity Assay by the Comet Assay

The cells were treated as in the MTT assay with concentration at IC_50_ value or at a maximum-treated concentration, in case no IC_50_ value was detected. The alkaline comet assay was used to assess the genotoxicity of plant extracts, according to a method previously described by Singh et al. [13]. Briefly, the electrophoresis buffer consisted of 0.3 M NaOH and 1 mM EDTA (pH = 10). The power was supplied at a constant of 3.4 V/cm with an adjustment to 300 mA, for 25 min. To quantify the level of DNA damage, the extent of DNA migration was defined using the “Olive Tail Moment” (OTM), which is the relative amount of DNA in the tail of the comet multiplied by the median migration distance. The comets were observed at 200 magnifications and images were obtained using an image analysis system (Isis) attached to a fluorescence microscope (Nikon, Japan), equipped with a 560 nm excitation filter, 590 nm barrier filter, and a CCD video camera PCO (Germany). At least 150 cells (50 cells for each of triplicate slides) were examined for each experiment. The CASP software (Wroclaw, Poland) was used to analyze the OTM. The negative control was untreated cells, and the positive control was UV-treated cells. All experiments were in triplicate. The triplicate cultures were scored for an experiment. All values were expressed as the mean ± S.D. The nonparametric Mann–Whitney* U* test was used for statistical analysis of the comet assay results; statistical significance was set at *p* < 0.05.

## 3. Results

**Figure 1:**
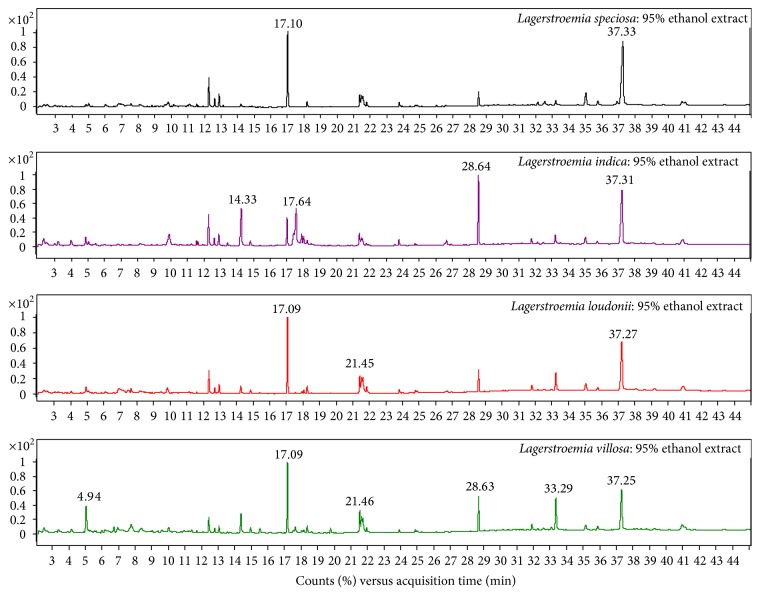
Chromatograms of ethanol crude extracts from the leaves of the three studied* Lagerstroemia speciosa*,* L. indica*,* L. loudonii*, and* L. villosa* species.

**Figure 2:**
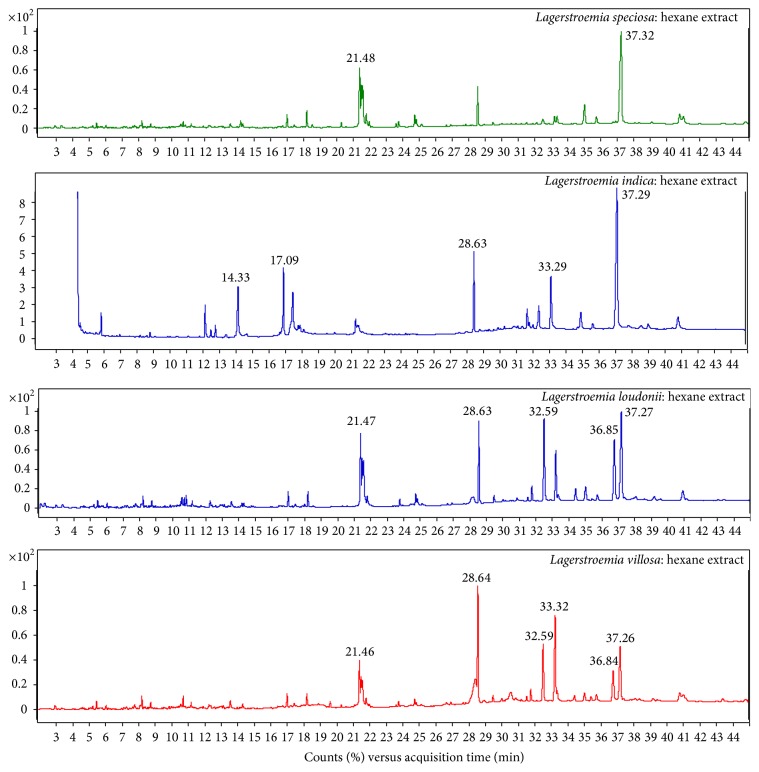
Chromatograms of hexane crude extracts from the leaves of* Lagerstroemia speciosa*,* L. indica*,* L. loudonii*, and* L. villosa*.

Phytochemical analysis of the filtrates from ethanol and hexane crude extracts (Figures 1 and 2) of the four studied samples as* L. speciosa*,* L. indica*,* L. loudonii*, and* L. villosa* revealed that there are several substances with some major components in higher amounts than others (Table 1). These are 34.4%  *γ*-sitosterol, 19.1% phytol, 34.3%  *γ*-sitosterol, and 27% (Z)-9-octadecenamide in* L. speciosa*; 13.5% squalene, 11.3% n-hexadecanoic acid, 11.2% linolenic acid, and 32.2%  *γ*-sitosterol in* L. indica*; 23.2%  *γ*-sitosterol, 18.4% phytol, 20.6% (Z)-9-octadecenamide, 18.4%  *γ*-sitosterol, 12.6% octacosane, and 12.4% tetratriacontane in* L. loudonii*; 16.9% phytol, 12.8% (Z)-9-octadecenamide, 18.2%  *α*-tocopherol, 16.2% (Z)-9-octadecenamide, 14.9% squalene, 14.7%  *γ*-sitosterol, and 11.3% octacosane in* L. villosa*, with ethanol and hexane solvents, respectively. Analysis of the plant extract component by HPLC actually concentrated on corosolic acid findings, and the results showed no detection with hexane in* L. speciosa* and* L. loudonii* and a very small amount in the other studied species (Table 2).

Mass of the crude extracts of the three samples derived from ethanol and hexane solvents is shown in Table 3. The extracts were subjected to serial 10-fold dilution for five levels, as used for the MTT assay.

**Figure 3:**
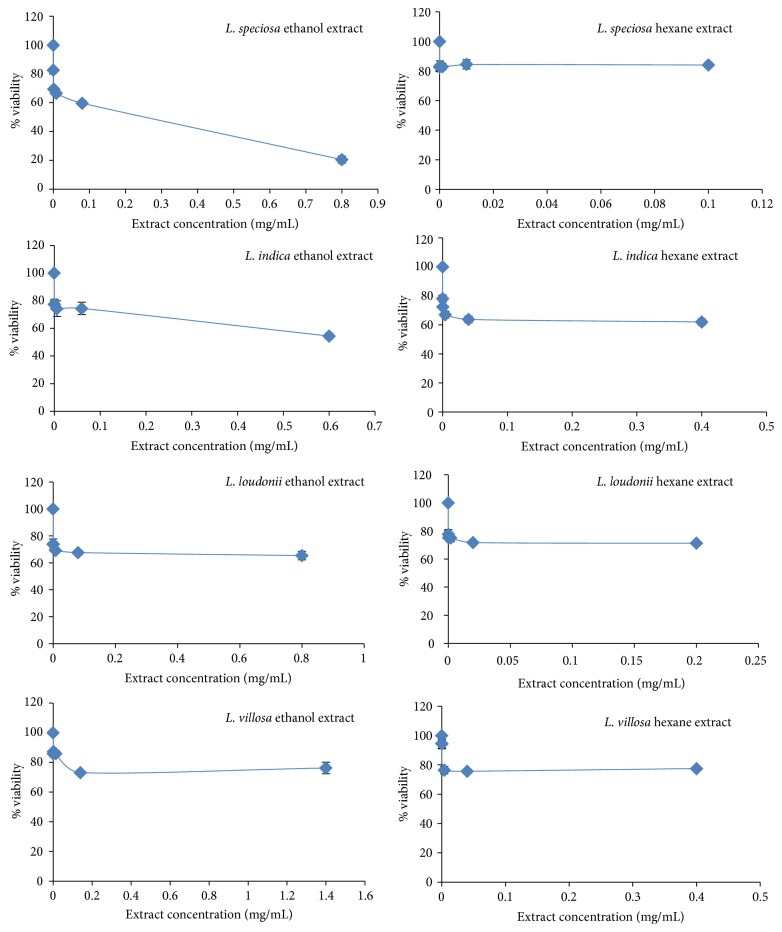
Cytotoxicity and IC_50_ values of ethanol and hexane extracts from the leaves of* Lagerstroemia speciosa*,* L. indica*,* L. loudonii*, and* L. villosa.*

The percentages of cell viability are 82.5 ± 2.5 to 84.5 ± 3.1 with hexane* L. speciosa* extract; 54.40 ± 2.15 to 77.46 ± 0.90 and 62.02 ± 2.20 to 78.15 ± 2.41 with ethanol and hexane* L. indica* extracts, respectively; 67.62 ± 1.82 to 73.83 ± 3.85 and 71.27 ± 0.72 to 77.60 ± 3.38 with ethanol and hexane* L. loudonii* extracts, respectively; and 73.18 ± 0.23 to 87.24 ± 1.17 and 75.67 ± 0.35 to 94.72 ± 3.74 with ethanol and hexane* L. villosa* extracts, respectively (Table 3, Figure 3). There is an IC_50_ value, 0.24 mg/mL, of ethanol* L. speciosa* extract, which refers to an LD_50_ of 811.78 mg/kg.

**Figure 4:**
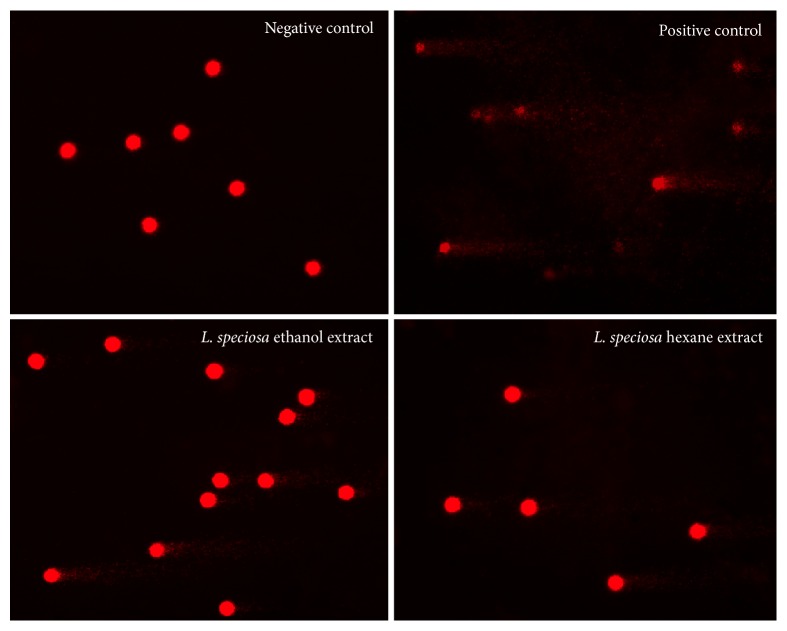
Comet assay images of PBMCs (200x); negative control, positive control, and examples of the extracted treatment, ethanol, and hexane extracts from the leaves of* Lagerstroemia speciosa*. Similar figures are not presented.

Because the ethanol* L. speciosa* extract and the ethanol and hexane* L. indica*,* L. loudonii*, and* L. villosa* extracts have no IC_50_ values and high % cell viability, the first highest diluted concentration extracts were selected for further step genotoxicity study as the comet assay. The results showed that, compared to negative control (untreated cells), the four tested species induced significant DNA damage in PBMCs (*p* < 0.05) (Table 4 and Figure 4).

## 4. Discussion 

Since the announcement that* L. speciosa* and* L. indica* contain corosolic acid, which is used in the prevention and treatment of type 2 diabetes [3, 6–9], the species studied here have been widely used in both prepared and traditional forms worldwide. Conversely, this research found a large amount of *γ*-sitosterol (14.7–34.4%) in all four of the studied species. Through GC-MS supported information by HPLC, lack or a small amount (0.002–0.07 mg/mL) of corosolic acid was detected. The quantity found leads to an assumption that corosolic acid may not be a factor in the treatment of diabetes. Currently, *γ*-sitosterol, an epimer of *β*-sitosterol, has been insisted to possess antihyperglycemic activity by increasing insulin secretion in response to glucose confirmed with immune histochemical study of pancreas [14, 15]. Additionally, Sundarraj et al. [16] demonstrated in vitro results that support the ethnomedical use of *γ*-sitosterol against cancer through the growth inhibition and cell cycle arrest on the apoptosis of cancer cells in accord with Endrini et al. [17], which showed that *γ*-sitosterol was cytotoxic against colon and liver cancer cell lines and that this effect was mediated by downregulation of c-myc expression and induction of the apoptotic pathways. Currently, studies in the many plant species where *γ*-sitosterol is found, such as in* Girardinia heterophylla* [18] and* Lippia nodiflora* [14], agree with the four studied* Lagerstroemia* species, the highest level found in* L. speciosa* and followed by the level in* L. indica*. The other substances in small amounts were quoted as phytol, (Z)-9-octadecenamide (oleamide), squalene, n-hexadecanoic acid, linolenic acid, octacosane, tetratriacontane, and *α*-tocopherol, most of which are beneficial in humans; for examples, oleamide is a protective agent against scopolamine-induced memory loss and is suggested as useful as a chemopreventive agent against Alzheimer's disease [19], and it induces deep sleep [20] and the upregulation of appetite [21, 22]. Squalene is a triterpene necessary for life. In the human body, it is a natural and essential component used for the syntheses of cholesterol, steroid hormones, and vitamin D. It may also be an anticancer substance, as it possesses chemopreventive activity [23, 24]. Phytol is a diterpene alcohol that can be used as a precursor for the manufacture of synthetic forms of vitamin E [25] and vitamin K1 [26] and is used in the fragrance industry and in cosmetics, shampoos, toilet soaps, household cleaners, and detergents. Its worldwide use has been estimated to be approximately 0.1–1.0 metric tons per year [27]. Hexadecanoic acid or palmitic acid and linolenic acid are types of fatty acids. Octacosane is an alkane, which has been used as a lubricant, transformer oil, and anticorrosion agent; parts of the paraffin or wax are chemically inactive (http://chemicalland21.com/industrialchem/organic/n-OCTACOSANE.htm). Each phytochemical actually has specific functions, but they may potentially not be known at all. Therefore, the tests for total substance contents, for human safety usage without toxicity, are further experiments of cytotoxicity and genotoxicity levels.

The mass showed higher concentration with ethanol solvent than hexane in all four studied species (Table 3). These assumptions are caused by the fact that polar phytochemicals dissolve more easily in ethanol because it is a more polar substance than the hydrocarbon hexane, which is part of the nonpolar group. The vehicle control (DMSO) was performed for every tested concentration, and it was demonstrated that DMSO does not induce cell death at the highest tested concentration (10%) in PBMCs, so the effects mentioned above can only be attributed to the plant extracts' bioactive compounds (data not shown). Therefore, it was not a surprise that IC_50_ with cytotoxicity appeared in the ethanol* L. speciosa* extracts, but not in the hexane extracts, when the same species were studied.

The MTT assay led to a LD_50_ at 811.78 mg/kg. The extrapolated data on predicted LD_50_ dose demonstrated that all tested compounds of* L. speciosa* belong to the WHO Class III (over 500 mg/kg body weight, oral), slightly hazardous category of toxic chemicals. For the evaluation of toxicity, 50 kg body weight would have to consume possibly a dose of 25,000 mg, to reach this level. However, consumers should see more toxicity by the in-depth comet assay. The first highest 10-fold diluted concentration extracts were selected for comet assay for the following reasons: firstly, to have the nearest concentration at usually human consuming of plant parts and secondly, to have not used more than 10% DMSO concentration for final 1% concentration, to avoid affecting on cells.

The results showed that, compared to negative control (untreated cells), the four tested species induced significant DNA damage in PBMCs (*p* < 0.05). Untreated cells for the negative control appeared as spherical nucleoids with no DNA migration. In the case of the positive control (UV-lighted cells), the gradual increase of strand breaks was evident, and they were represented as cells with a long tail of DNA streaming out from the nucleoid, forming a comet-like appearance (Figure 4).

## 5. Conclusion

The phytochemical *γ*-sitosterol found in high amount in the two studied species,* L. speciosa* and* L. indica,* was very interesting, but consumers should consider toxicity of the plants.

## Figures and Tables

**Table 1:** Chemical constituents of ethanol and hexane extracts of *Lagerstroemia speciosa*, *L. indica*, *L. loudonii,* and *L. villosa*.

Compound	Formula	Relative content (%)							
		*L. speciosa* (ethanol)	*L. speciosa *(hexane)	*L. indica *(ethanol)	*L. indica *(hexane)	*L. loudonii *(ethanol)	* L. loudonii *(hexane)	*L. villosa *(ethanol)	*L. villosa* (hexane)
*γ*-Sitosterol	C_29_H_50_O	34.44	34.34	21.91	32.18	23.22	18.39	17.90	14.70
(Z)-9-Octadecenamide (oleamide)	C_18_H_35_NO	9.73	26.99	4.98	4.04	14.07	20.60	12.75	16.24
Phytol	C_20_H_40_O	19.10	1.93	5.24	7.31	18.44	1.51	16.89	1.71
*α*-Tocopherol	C_29_H_50_O_2_	1.23	1.66	1.57	8.01	5.22	7.48	10.52	18.24
Squalene	C_30_H_50_	3.06	5.99	13.54	9.24	5.15	8.74	8.42	14.89
Octacosane	C_28_H_58_	—	—	—	—	—	12.63	—	11.31
Tetratriacontane	C_34_H_70_	—	—	—	—	—	12.39	—	7.53
n-Hexadecanoic acid	C_16_H_32_O_2_	0.90	0.90	11.29	7.40	1.93	—	5.18	—
Linolenic acid	C_18_H_32_O_2_	—	—	11.19	6.36	—	—	—	—
5-Hydroxymethylfurfural	C_6_H_6_O_3_	0.60	—	1.98	—	1.61	—	8.54	—
Phytol, acetate	C_22_H_42_O_2_	6.51	—	5.60	3.16	5.70	0.62	3.65	—
Campesterol	C_28_H_48_O	5.09	5.72	1.68	3.12	2.96	2.69	2.09	1.92
Ethyl *α*-d-glucopyranoside	C_8_H_16_O	2.44	—	5.27	—	2.40	—	1.62	—
3,7,11,15-Tetramethyl-2-hexadecen-1-ol	C_20_H_40_O	4.83	—	3.25	1.89	3.46	—	2.05	—
1,2,3-Benzenetriol	C_13_H_18_O_4_	1.37	—	—	—	3.82	—	—	—
Linoleic acid	C_18_H_32_O_2_	—	—	3.74	1.83	—	—	—	—
Vitamin E^*∗*^	n/a	1.74	3.50	—	—	—	—	3.13	1.35
Stigmastan-3,5-diene	C_29_H_48_	—	—	—	3.30	—	—	—	—
24-Methylenecycloartanol	C_31_H_52_O	—	—	2.09	3.04	3.27	2.16	—	—
cis-11-Eicosenamide	C_20_H_39_NO	0.41	3.25	—	—	0.60	2.00	0.79	0.71
Stigmast-5-en-3-ol,oleate	C_47_H_82_O_2_	—	3.07	—	—	—	—	—	—
*γ*-Tocopherol	C_28_H_48_O_2_	—	—	1.00	2.65	1.35	1.76	1.42	1.88
Hexadecanamide	C_16_H_33_NO	1.25	2.52	0.55	0.31	1.79	1.58	1.57	1.66
Tetratetracontane	C_44_H_90_	—	—	—	—	—	1.83	—	—
Octadecanamide	C_18_H_37_NO	1.06	1.80	0.35	0.15	1.18	0.66	0.62	0.69
*α*-Tocopherolquinone	C_29_H_50_O_3_	—	1.78	—	—	—	0.86	—	1.53
Methane, tris(methylthio)-	C_4_H_10_S_3_	—	—	—	1.77	—	—	—	—
Octadecanoic acid	C_18_H_36_O_2_	—	—	1.69	0.40	—	—	—	—
Stigmasterol	C_29_H_48_O	1.40	1.65	0.44	0.91	1.00	0.89	1.19	1.10
Octadecane	C_18_H_38_	—	0.87	—	—	—	0.96	—	1.30
Cycloartenol	C_30_H_50_O	—	—	—	1.16	—	—	—	—
Glycerol *β*-palmitate	C_19_H_38_O_4_	1.00	0.93	0.88	—	0.91	0.71	0.73	0.64
17-Pentatriacontane	C_35_H_70_	0.96	—	—	—	—	—	—	—
2-Methoxy-4-vinylphenol	C_9_H_10_O_2_	0.94	—	—	—	—	—	—	—
Lupeol	C_30_H_50_O	—	—	—	—	—	0.94	—	—
Hexadecanoic acid, ethyl ester	C_16_H_32_O_2_	—	—	0.87	—	0.76	—	0.94	—
Pentacosane	C_25_H_52_	—	0.50	—	—	—	—	—	0.92
Dodecane, 4,6-dimethyl-	C_14_H_30_	—	0.68	—	—	—	0.60	—	0.90
Linolenic acid, ethyl ester	C_18_H_30_O_2_	—	—	0.89	0.62	0.75	—	—	—
1,30-Triacontanediol	C_30_H_62_O_2_	—	—	—	—	—	—	—	0.78
Cholesta-4,6-dien-3-ol	C_27_H_44_	0.78	—	—	—	—	—	—	—
Tetracosane	C_24_H_50_	—	0.77	—	—	—	—	—	—
1,2-Propanediol, 3-(1-pyrrolidinyl)-	C_7_H_15_NO_2_	0.72	—	—	—	—	—	—	—
Tricosane	C_23_H_48_	—	0.70	—	—	—	—	—	—
Docosane	C_22_H_46_	—	0.45	—	—	—	—	—	—
9,12-Octadecadienoic acid, ethyl ester	C_18_H_32_O_2_	—	—	—	—	0.41	—	—	—
Dihydroactinidiolide	C_11_H_16_O_2_	—	—	—	0.34	—	—	—	—
Olean-12-en-3-one	C_30_H_48_O	—	—	—	0.32	—	—	—	—
5-Methoxy-2-oxoestra-1(10),3-dien-17-yl acetate	C_21_H_28_O_3_	0.30	—	—	—	—	—	—	—
7-Dehydrodiosgenin	C_27_H_40_O_3_	—	—	—	0.22	—	—	—	
Diisooctyl phthalate	C_24_H_38_O_4_	—	—	—	0.16	—	—	—	—
*α*-Hydroxymyristic acid	C_14_H_28_O_3_	0.14	—	—	—	—	—	—	—
*α*-Glyceryl linoleate	C_21_H_36_O_4_	—	—	—	0.11	—	—	—	—

^*∗*^Vitamin E refers to a class of compounds; n/a: not analyzed as individual compound by the Wiley 7N.1 library.

**Table 2:** The contents of corosolic acid (mg/mL) determined by HPLC from leaf extracts of *Lagerstroemia *species.

Plant samples	Amount in each type of solvent (mg/mL)	
	Ethanol	Hexane
*L. speciosa*	0.068	Not detected
*L*.* indica*	0.0036	0.0015
*L*. *loudonii*	0.093	Not detected
*L*. *villosa*	0.125	0.0012

**Table 3:** Mass concentration with ethanol and hexane solvents, IC_50_ values, and % cell viability of the three studied* Lagerstroemia speciosa*, *L. indica L. loudonii,* and *L. villosa *species.

Plant	Solvent	Maximum extract conc. (mg/mL)	IC_50_ (mg/mL)	% cell viability
*L. speciosa *	Ethanol	8	0.24	—
	Hexane	1	—	82.54 ± 2.52–84.45 ± 3.11

*L. indica*	Ethanol	6	—	54.40 ± 2.15–77.46 ± 0.90
	Hexane	4	—	62.02 ± 2.20–78.15 ± 2.41

*L. loudonii*	Ethanol	8	—	67.62 ± 1.82–73.83 ± 3.85
	Hexane	2	—	71.27 ± 0.72–77.60 ± 3.38

*L. villosa *	Ethanol	14	—	73.18 ± 0.23–87.24 ± 1.17
	Hexane	4	—	75.67 ± 0.35–94.72 ± 3.74

**Table 4:** The level of DNA damage expressed as Olive Tail Moment (OTM) in PBMCs after treatment with ethanol and hexane *Lagerstroemia speciosa*, *L*. *indica*, *L*. *loudonii,* and *L*. *villosa* leaf extracts with the first 10-fold dilution concentrations selected.

Plant	Solvent	Concentration (mg/mL)	Olive tail moment	*P* value
*L. speciosa*	Ethanol	0.24	0.21 ± 0.22	<0.0001
	Hexane	0.10	0.42 ± 0.28	<0.0001
	Negative control	—	0.02 ± 0.03	—

*L*. *indica*	Ethanol	0.60	3.84 ± 2.91	<0.0001
	Hexane	0.40	1.28 ± 1.24	<0.0001
	Negative control	—	0.39 ± 0.36	—

*L*. *loudonii*	Ethanol	0.80	0.66 ± 0.55	<0.0001
	Hexane	0.20	0.45 ± 0.35	0.0228
	Negative control	—	0.39 ± 0.36	—

*L*. *villosa*	Ethanol	1.40	1.20 ± 0.50	<0.0001
	Hexane	0.40	0.60 ± 0.67	<0.0001
	Negative control	—	0.39 ± 0.36	—
